# A comparative study of acute-phase protein concentrations in historical and modern broiler breeding lines

**DOI:** 10.3382/ps/pey272

**Published:** 2018-06-30

**Authors:** E L O’Reilly, R A Bailey, P D Eckersall

**Affiliations:** 1 Institute of Biodiversity, Animal Health & Comparative Medicine, University of Glasgow, Bearsden Rd, Glasgow G61 1QH, UK; 2 Aviagen Ltd., Lochend Road, Newbridge, Midlothian EH28 8SZ, UK

**Keywords:** broiler, acute-phase protein, lineage, innate immune system

## Abstract

Acute-phase proteins (APP) are secreted from the liver as a result of inflammation or infection and are measurable in serum and plasma. To determine whether the constitutive APP serum amyloid A (SAA), alpha-1-acid glycoprotein (AGP), ceruloplasmin (Cp), and ovotransferrin (Ovt) have changed as a result of selection for improved production and growth characteristics over the last 40 yr two historical broilers lines were compared to a modern line of the same lineage. Serum was harvested from blood samples taken from the 3 broiler lines on days 10, 17, and 20, and the APP concentrations were determined using immunoassay methods. Most of the significant changes observed were age related, with SAA and Cp having significantly lower concentrations at day 20 than days 10 and 17 in all lines. The only significant difference between lines was observed at day 20 on which both Cp (*P* = 0.01) and AGP (*P* = 0.03) were significantly higher in the modern line than the 90s line, though no significant differences were noted between the modern and 70s line. When evaluating the difference in APP concentrations between males (Cx) and females (Px) across all 3 lines, females had a higher SAA at day 17 and lower SAA at day 20, *P* = 0.0078 and 0.0327 respectively, and males had a significantly higher Ovt on days 17 and 20 (*P* = 0.0002 and *P* = 0.003 respectively). These results reveal that APP concentrations fluctuate over this early period of growth and that the changes in APP serum concentration appear uniform between 3 lines with very contrasting selection history, suggesting the improvements made in meat production efficiency since the 1970s have not affected the circulating concentrations of these constitutively expressed APP.

## INTRODUCTION

The systemic acute-phase response (**APR**) is the inducible, non-specific component of the innate immune response. It is the systemic reaction to local or systemic disturbances caused by trauma, infection, stress, surgery, neoplasia, or inflammation the goal of which is re-establishment of homeostasis and healing (Klasing, 2004; Gruys et al., 2005; Cray et al., 2009; O’Reilly and Eckersall, 2014). Within the first few hours of an APR, protein synthesis within the liver and hepatocyte secretion is drastically altered, and there are measureable changes in the plasma concentration of several plasma proteins referred to as acute-phase proteins (**APP**) (Gruys et al., 2005). Hepatic mRNA synthesis of the APP that increases during an APR is upregulated, and there is a reduction of mRNA synthesis of those APP that decrease during an APR (Gruys et al., 2005). These are termed positive APP or negative APP. Positive APP are further classified as minor, moderate, or major APP according to the magnitude and duration of increase during the APR. Major APP increase 10 to 1,000-fold, moderate APP increase 4 to 10-fold and minor APP represent those with only slight 2 to 3-fold increases (O’Reilly and Eckersall, 2014). There are noted differences in the timing and duration of increase in positive APP. Major APP tend to increase markedly within the first 48 h after of the triggering event and decline rapidly due to a short half-life. Moderate and minor proteins tend to increase more slowly and have more prolonged duration (Cray et al., 2009; O’Reilly and Eckersall, 2014).

Constitutively expressed APP are present during the homeostatic state, circulating at basal levels that are measurable, usually by immunoassay. The constitutive presence of APP indicates involvement in crucial metabolic and immune pathways during a normal healthy homeostatic state. During an APR their increased concentration serves to return the animal to homeostasis by mediating a range of metabolic and immune processes such as opsonization, complement activation, binding of cellular remnants, neutralizing enzymes, scavenging free hemoglobin, iron and free radicals and exhibiting antibacterial, antiviral and antioxidant functions (reviewed in O’Reilly and Eckersall, 2014).

In chickens ovotransferrin (**Ovt**) and alpha-1-acid glycoprotein (**AGP**) are moderate APP (Takahashi et al., 1994; Xie et al., 2002) and both are constitutively present and measurable in serum between 1 and 1.5 g/L and 0.15 and 0.25 g/L, respectively (Inoue et al., 1997; Takahashi et al., 1998; Durairaj et al., 2009; Shakeri et al., 2014). In chickens, serum amyloid A (**SAA**) appears to be the only major APP. Although studies investigating the response of birds infected with infectious bursal disease virus (**IBDV**) and infectious bronchitis virus have shown only mild 1.5 to 2-fold increases in SAA (Nazifi et al., 2010, 2011; Seifi et al., 2014), earlier work found SAA to increase 100 to 1,000-fold 12 h post-injection as a result of turpentine and *Staphylococcus aureus* injection (Upragarin, 2005). The difference in response may be attributable to the eliciting cause or the immunoassay methods employed; it is notable that the earlier report by Upragarin (2005) of large increases in SAA concentration used chicken-specific antibodies. In chickens, ceruloplasmin (**Cp**) is constitutively present, but at lower concentrations than observed in mammals (Disilvestro and Harris, 1985). Cp is considered a minor APP in chickens, with basal concentrations ranging from 0.03 to 0.12 g/L, increasing 2 to 3-fold during an APR (Disilvestro and Harris, 1985; Richards and Augustine, 1988; Georgieva et al., 2010; Najafi et al., 2015a).

During an APR, the magnitude of change of circulating concentrations of APP is usually related to the severity of initiating cause or disorder and the extent of tissue damage. Quantification of their concentration can therefore provide diagnostic and prognostic information and assessment of the response to the triggering event (Murata et al., 2004). The changes in the APP profile of an animal during an APR also represent an immune parameter by which the degree of immune com petence could be measured. High levels of circulating APP may not necessarily be optimal and desirable because, as effective as the APR is, it is costly in terms of energy expenditure and behavioral changes (Klasing, 2004; Kogut, 2009). Studies in physiological ecology and theoretical immunology often refer to “cost” in terms of resources such as the energy needed to mount an immune response, or trade-offs between immunocompetence and other nutrient requiring functions (Read and Allen, 2000). The systemic APR that accompanies the innate immune response appears to be the most expensive component of immunity in young chickens (Klasing, 2004) with the APR associated with depressed performance in this species (Peebles et al., 2014).

Advances in animal breeding, genetic selection, nutrition, management, and disease control have facilitated the advancement in scale of production of the broiler chicken (*Gallus gallus*). Through these advances, there have been improvements in the feed conversion ratio allowing for improved growth rates and an increase in breast yield. There have been reports suggesting that selection for biological efficiency has had an adverse effect on immune competence (Swaggerty et al., 2009). Although it has been demonstrated that measurable differences in innate responsiveness is under genetic control with strong pro-inflammatory cytokine and chemokine responses associated with increased resistance against disease (Swaggerty et al., 2009), there have been no studies looking at APP in different chicken lines. The effect of selective breeding on the many elements of the birds immune response has been the subject of discussion and research for many years (Cheema et al., 2007; Hocking, 2010). Here, we report the APP concentrations of 4 positive APP at 3 time points between days 10 and 20 in 3 pure bred pedigree chicken lines of the same lineage. These lines include on-selected historic lines from the 1970s (70s), the 1990s (90s) and a modern line that has been selected for broad breeding goals including biological efficiency, skeletal and cardiovascular fitness, fertility, hatchability, and egg production. It was the objective of this study to compare constitutive APP concentrations of the 2 historical and a modern line of broilers over this period of growth to determine whether selective breeding over a 40 yr period has resulted in any difference in the basal concentrations of these APP.

## METHODS

### Experimental Design

The 70s, 90s, and modern broiler lines were all housed within an environmentally controlled broiler farm in southern Scotland. The birds were all housed in pens with wood shavings provided as the litter substrate with *ad libitum* access to food and water. The stocking densities for the birds were between 29 and 32 kg per m^2^ in line with the guidelines set down in the EU Council Directive 2007/43/EC. All birds were incubated in the same hatchery, where they received infectious bronchitis vaccine (Poulvac IB Primer Zoetis, according to the manufacturers recommendations) and were tagged with a barcoded wingband for identification. Once hatched, the birds were moved all together to the growing farms where they were placed in the same poultry house in pens according to line. Birds were vaccinated for turkey rhinotracheitis virus (**TRT**) (Nemovacc, Merial) and IBDV (AviPro Gumboro, Lohman Animal Health), in-line (water) as per manufacturers recommendations on day 12 and on days 15 and 16, respectively. Blood for APP analysis was recovered by venipuncture of the wing vein and collected into 2 ml polypropylene serum tubes (Ambion ThermoFisher Scientific, AM12475, Renfrew, Scotland) on day 10 (prior to on farm vaccination) and days 17 and 20 (post on farm vaccination) from the 70 s line (n = 9, 10 and 9 respectively), 90 s line (n = 10 at all-time points) and modern (n = 9, 10, and 10 respectively). Blood was allowed to clot at room temperature, before being transferred to the laboratory on ice where the serum was recovered and stored at −80°C until use.

### APP Measurement

Following further in house validation (Kaab et al., 2018), the concentrations of SAA, AGP, and Cp were determined using ELISAs (3400-7, 2510-3, 2610-3: Life Diagnostics, West Chester, PA) and in a deviation from the manufacturers recommendation whole serum samples were diluted 20-fold for SAA and 40,000 fold for Cp. For AGP, the manufacturer recommendations were followed and a 10-fold dilution was utilized. Ovotransferrin concentration was determined using a radial immunodiffusion assay that utilizes neat serum and sheep anti-chicken Ovt antibody raised against purified egg Ovt (Conalbumin Sigma C0755) (O’Reilly, 2016). GraphPad Prism v.5 and v.6 were used for statistical analysis. All results were analyzed using the Kruskal–Wallis test with the Dunn post-test analysis, with significance noted at *P* < 0.05.

## RESULTS AND DISCUSSION

**Figure 1.**
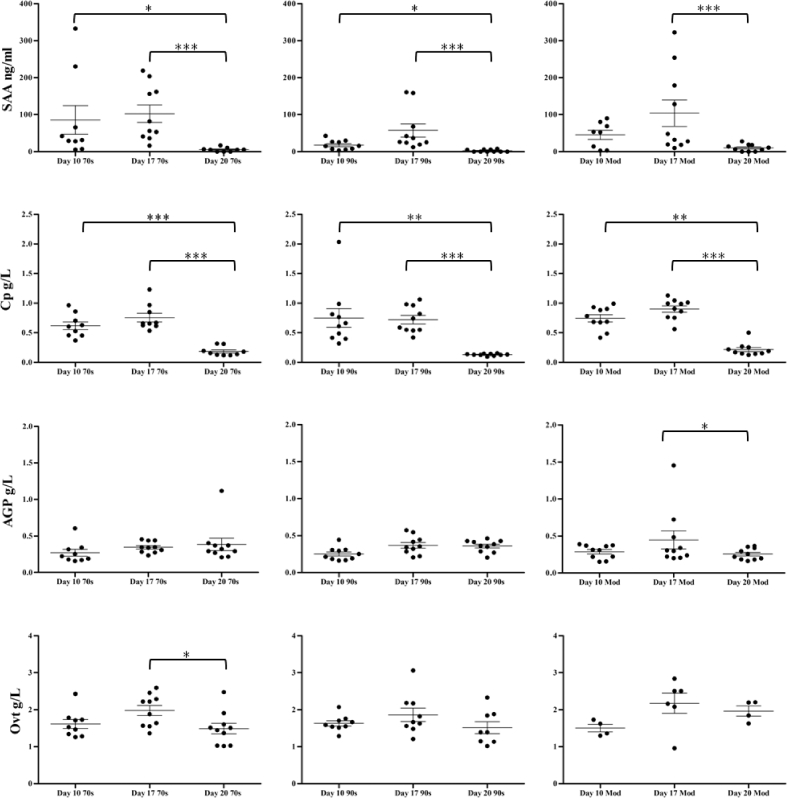
Serum concentration of serum amyloid A (SAA), alpha-1-acid glycoprotein (AGP), ceruloplasmin (Cp), and ovotransferrin (Ovt) in 3 lines of broiler on days 10, 17, and 20 (mean ± SEM) (*<0.05, **<0.01, ***<0.001).

Comparing APP concentrations of 2 historical lines from the 70s and the 90s with a modern broiler line was undertaken with the aim of determining whether the constitutive APP concentrations differ, and as such have been affected, by selection for production traits. Figure 1 details the serum concentration of the APP over the 3 time points, in the 3 lines. The majority of the significant changes in APP concentrations were observed over time in all 3 of the lines. Serum amyloid A was significantly different between each time point in the 70s and 90s lines (*P* = <0.0001) and the modern line (*P* = <0.01) with concentrations lower at day 20 than days 10 and 17 in all lines. Similarly, Cp was significantly different between each time point (*P* = <0.0001) with concentrations lower at day 20 than days 10 and 17 in all lines. For AGP, the changes over time were not as variable as the other APP, a significant difference only observed between days 17 and 20 in the modern line (*P* = <0.05). Previous work suggests that there is a normal increase in AGP concentration over the early period of growth with significant increases over time between days 12 and 22 (O’Reilly, 2013), though studies focusing on AGP between 3 and 7 wk of age found AGP not to be affected age (Inoue et al., 1997).

**Figure 2.**
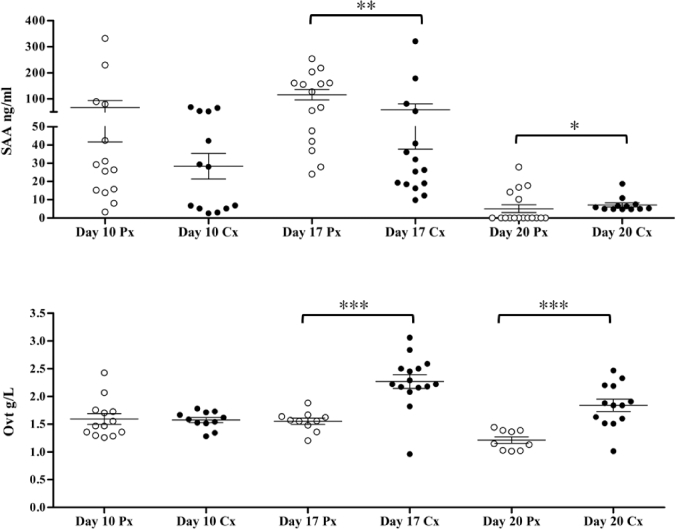
Scatter plot of SAA and Ovt concentrations in male (Cx) and female (Px) broilers over consecutive time points across all lines studied (70s, 90s, and modern) (Mean ±SEM , *<0.05, **<0.01, ***<0.001).

Ovotransferrin concentration was significantly higher at day 17 for the 70s line only. When evaluating the difference in APP concentrations between males and females, for both Ovt and SAA on days 17 and 20, there was significant difference, with females having higher SAA at day 17 and lower SAA at day 20, *P* = 0.0078 and *P* = 0.0327 (Figure 2). For Ovt the males had significantly higher Ovt on both days 17 and 20 (*P* = 0.0002 and *P* = 0.003 respectively) (Figure 2). The synthesis of Ovt in the liver is under hormonal regulation, with oestrogen having a regulatory action, increasing Ovt mRNA synthesis in the liver (Mcknight and Palmiter, 1979; Mcknight et al., 1980). At a protein level, females have been previously been shown to have higher serum Ovt concentrations than males (Saleem, 2013). It is possible that there are sex-linked differences in the innate immune response as it has previously been shown that the sire is more influential in determining heterophil functional efficiency (Swaggerty et al., 2009). Differences in growth rates between the sexes could also influence the synthesis of constitutive APP such as Ovt.

Although significant differences were readily identifiable between the time points in 3 lines and between the sexes, few were identified between the lines. All the lines follow the same trend over time with significant between line difference identified only at day 20 for Cp (*P* = 0.01) and AGP (*P* = 0.03). Post-test analysis revealed the significance to lie specifically between the 90s line and the modern line, the modern line having higher Cp and AGP than the 90s line, though no significant difference was noted between the modern and 70s lines. Other studies evaluating the APR between chickens lines have found increased mRNA expression levels of pro-inflammatory cytokines in heterophils isolated from the more resistant lines compared to the susceptible lines (Swaggerty et al., 2009). Heterogeneity in phagocytic and bactericidal activities of heterophils have been observed, with chicken lines with functionally less active heterophils more susceptible to infections than those with highly functional heterophils (Swaggerty et al., 2003, 2009; Ferro et al., 2004). The selection of layers for high and low group productivity and survivability resulted in significantly higher percentages of blood lymphocytes and CD4+: CD8+ ratios of circulating T cells and significant differences in hematological parameters including heterophil: lymphocyte ratio (Cheng et al., 2001). For APP, few studies have been undertaken to compare the expression of APP between lines of chickens, with mannan binding lectin the only APP to be studied in this way with selection for high or low expression of mannan binding lectin producing chicken strains with high or low expression of this APP (Laursen et al., 1998); this study demonstrating, for mannan binding lectin at least, that expression is under genetic control. The results of the current study suggest that during the last 4 decades of genetic selection to improve body weight, growth rate, and feed conversion ratio, the constitutive concentrations of the 4 APP measured have been largely unaffected. The production of APP is costly in terms of nutrition and growth as APP are secreted in large amounts by the liver and quantitatively the APR is markedly more costly than the subsequent adaptive response (Iseri and Klasing, 2013). The production of APP is nutritionally costly as a significant amount of nutrients is needed to support their *de novo* synthesis (Klasing, 2004). The mobilization of skeletal muscle protein also occurs during immune stress response to supply the necessary amino acid precursors for APP synthesis (Liu et al., 2015). Given the energetic requirements of APP production, their constitutive presence under healthy normal conditions is evidence of an important role in maintaining the health of the bird.

The effect, if any of vaccination in this study, is not clear. Vaccinations for TRT and IBDV, delivered on days 12 and 15/16, respectively, could potentially account for the higher concentrations of SAA and Cp at day 17 and the subsequent decrease by day 20, but do not account for the higher levels seen at day 10. Previous work has found AGP (Inoue et al., 1997; Kaab et al., 2018) and SAA (Kaab et al., 2018) to increase in response to vaccination in chickens, with the increased AGP noted where IBDV vaccination was associated with the presence of lesions (Inoue et al., 1997). The presence and subsequent decrease in maternally derived antibody may possibly account for the significant changes seen over time in all 3 lines, particularly the increase between days 10 and 17. Like all vertebrates, hens will pass on antibody representative of their cummative antigen exposure prior to egg laying (Lemke et al., 2003). Antibodies derived from vaccination as well as antibodies to endemic pathogens that hens have in circulation will be transmitted to the offspring providing the young with protection (Grindstaff, 2008). As most maternally derived antibodies for vaccinated parent flocks are depleted by day 10 (Gharaibeh and Mahmoud, 2013), it is possible that a normal, protective, and non-pathological rise in constitutive APPs occurs as the maternally derived antibody declines.

Comparing the values from this study to previous studies reveals that SAA and AGP concentrations fall within and below the reported basal concentrations for these APP (Inoue et al., 1997; Takahashi et al., 1998; Alasonyalilar et al., 2006; Nazifi et al., 2010). Ovotransferrin, a widely measured APP has reported basal concentrations ranging from 0.2 to 1.18 g/L (Rath et al., 2009; Najafi et al., 2015a), lower than what was observed in this study across all of the lines (Table 1). Similarly, control concentrations of Cp have been previously reported as 0.0118 to 0.05 g/L (Richards and Augustine, 1988; Georgieva et al., 2010), much lower than the concentrations observed in this study (Table 1). Although this study provides further baseline APP values in birds from 10 to 20 d, it also serves to highlight the lack of data and information relating to healthy and normal constitutive APP concentrations across a wider age range that encompasses the productive life of broilers and broiler breeders. Although APP values from healthy chickens have been previously collated and reported by evaluating the control groups from the many experimental studies on APP from the 1960s onwards (O’Reilly and Eckersall, 2014; O’Reilly, 2016), attempting to compare and utilize such data as a reference range is problematic. These studies span a considerable period of time and utilize different types of birds of varying ages. The variation in assay type and limited availability of validated, chicken-specific assays also makes any meaningful comparisons difficult.

**Table 1.** Descriptive statistics of the APP concentrations SAA (ng/l), AGP, Cp, and Ovt (g/L) of the 70s, 90s and modern lines over 3 time points (standard error row removed)

APP		Day 10			Day 17			Day 20		
		70s	90s	Mod	70s	90s	Mod	70s	90s	Mod
N		9	10	10	9	10	10	9	10	10
SAA	Minimum	5.20	3.02	2.56	16.24	12.30	9.89	0.00	0.00	0.00
	Maximum	332.40	42.43	89.48	218.80	160.50	321.70	16.77	7.51	27.89
	Median	31.15	15.54	52.84	68.43	31.61	39.97	4.92	0.00	8.69
	Mean	85.63	17.81	45.40	102.20	57.22	103.90	5.38	2.36	10.11
	Std. Deviation	115.20	12.81	34.60	75.13	55.93	112.30	5.51	3.12	9.51
Cp	Minimum	0.37	0.32	0.42	0.54	0.42	0.56	0.12	0.09	0.13
	Maximum	0.96	2.03	0.99	1.23	1.06	1.13	0.32	0.15	0.50
	Median	0.60	0.64	0.73	0.66	0.66	0.95	0.16	0.13	0.18
	Mean	0.62	0.75	0.74	0.76	0.72	0.90	0.18	0.13	0.22
	Std. Deviation	0.20	0.50	0.19	0.22	0.22	0.17	0.08	0.02	0.11
AGP	Minimum	0.16	0.16	0.15	0.23	0.21	0.20	0.21	0.20	0.16
	Maximum	0.60	0.44	0.39	0.45	0.57	1.45	1.12	0.46	0.37
	Median	0.22	0.23	0.31	0.34	0.35	0.30	0.31	0.37	0.24
	Mean	0.27	0.25	0.29	0.35	0.37	0.45	0.39	0.36	0.26
	Std. Deviation	0.14	0.09	0.09	0.08	0.12	0.39	0.26	0.08	0.08
Ovt	Minimum	1.26	1.28	1.30	1.36	1.20	0.96	1.01	1.01	1.63
	Maximum	2.42	2.07	1.73	2.59	3.06	2.84	2.47	2.33	2.20
	Median	1.53	1.59	1.49	2.05	1.67	2.33	1.48	1.39	2.02
	Mean	1.61	1.63	1.50	1.98	1.86	2.17	1.49	1.52	1.97
	Std. Deviation	0.36	0.21	0.21	0.43	0.55	0.65	0.45	0.46	0.28

The current study, revealing that APP concentrations of 2 of the 4 APP measured varied significantly between 10 and 20 d, further highlights the indication for studies to fully characterize the normal values of APP over the full production life of broilers, particularly as their measurement and application in various areas of poultry science continues to increase. Also the studies on various infectious disease challenges, an area covered extensively in the literature, APP measurement in other areas including vaccination (Sylte and Suarez, 2012; Peebles et al., 2014), housing and production system (Tuyttens et al., 2008; Salamano et al., 2010), nutrition (Takahashi et al., 1995, 2002; 2009) and welfare (Najafi et al., 2014, 2015a, b) reveal the wide application for their measurement across the poultry sciences. Also shedding further light on the acute-phase element of the early innate responses, other potential stressors or factors that could affect the production of constitutive APP are being identified. An APR, characterized by increased serum concentrations of APP is undesirable for broiler production irrespective of the eliciting cause, be it infectious, inflammatory or vaccination. However, the energetic demands of producing constitutive APP, together with the significant changes during the period of growth characterized in this study, imply an important protective role in the innate responses of the chicken; the increased concentration of SAA and Cp at day 10 and 17 signifies particular importance at these ages particularly. The fact that these changes appear unchanged by selection further highlights the importance of these proteins. One of the key approaches taken by primary breeders is multi-environment selection (Kapell et al., 2012; Neeteson-van Nieuwenhoven et al., 2014) that allows health and welfare traits to be incorporated into selection in addition to the key production traits. The resultant improvements made in broiler performance through balanced breeding, do not appear however to have affected the constitutive concentrations of the APP SAA, AGP, Cp, or Ovt at this early stage of growth.
